# A case of endovascular treatment for acute portal vein thrombosis following portal vein resection and hepatectomy for hilar cholangiocarcinoma

**DOI:** 10.1093/bjrcr/uaaf017

**Published:** 2025-03-20

**Authors:** Sukru Oguz, Hakan Küçükaslan, Gokalp Altun, Dilek Basar, Serdar Topaloglu, Adnan Calik

**Affiliations:** Department of Radiology, School of Medicine, Karadeniz Technical University, Trabzon 61080, Turkey; Department of Surgery, Trabzon Akçaabat Haçkalı Baba Devlet Hastanesi, Trabzon 61080, Turkey; Department of Cardiovascular Surgery, School of Medicine, Karadeniz Technical University, Trabzon 61080, Turkey; Department of Pediatric Surgery, University of Health Sciences, Trabzon Kanuni Training and Research Hospital, Trabzon 61080, Turkey; Department of Surgery, School of Medicine, Karadeniz Technical University, Trabzon 61080, Turkey; Department of Surgery, School of Medicine, Karadeniz Technical University, Trabzon 61080, Turkey

**Keywords:** acute portal vein thrombosis, percutaneous portal vein stenting, portal vein reconstruction

## Abstract

Currently, portal vein (PV) resection is performed in 10%-40% of liver resections performed for hilar cholangiocarcinoma (HC). The defect is generally repaired with a patch of an autologous vein graft or end-to-end anastomosis after complete separation of the main PV trunk and the left PV. Postoperative PV thrombosis is a severe complication occurring in 2%-9% of patients requiring PV reconstruction. Here in, we presented a 55-year-old man with abdominal pain without hyperbilirubinaemia who was diagnosed with HC. The patient underwent right hepatectomy, extrahepatic biliary resection, and PV resection. The PV defect was repaired with autologous umbilical vein graft. Following the operation, acute PV thrombosis was encountered postoperative day 1. We conducted the treatment of the early acute PV thrombosis by intraportal tPA and PV stenting with endovascular approach.

## Introduction

Following improvements in surgical technique and perioperative care, portal vein (PV) resection is now performed in 10%-40% of liver resections for hilar cholangiocarcinoma (HC).^1^ Reports from high-volume centres indicate that PV resection has not been associated with an increase in morbidity or mortality at subgroup analysis.^2^ Partial resection of the remnant PV in the right or extended right hepatectomies (the left PV, the right and left PV bifurcation, or the main PV trunk) is easily performed due to the anatomical advantage posed by the left PV. The long extrahepatic length of the left PV facilitates surgical liberation. There is generally no need for interposition grafts when resecting the left PV. The defect is generally repaired with a patch of an autologous vein graft or end-to-end anastomosis after the complete separation of the main PV trunk and the left PV.^1^^,^^3^ However, the patency of the repair site is threatened by the discrepancy between the calibres of the main PV trunk and the left PV, altered tension in the anastomosis after fixation of the left liver lobe to the diaphragmatic remnant of the falciform ligament of the liver, or external compression from bilioenteric anastomosis. Postoperative PV thrombosis is a severe complication occurring in 2%-9% of patients requiring PV reconstruction and leads to postoperative hepatic failure (POHF) and death in most cases.^4^ We describe the management of acute PV graft occlusion following right hepatectomy and PV resection for HC with a percutaneous endovascular approach.

## Clinical presentation

**Figure 1.**
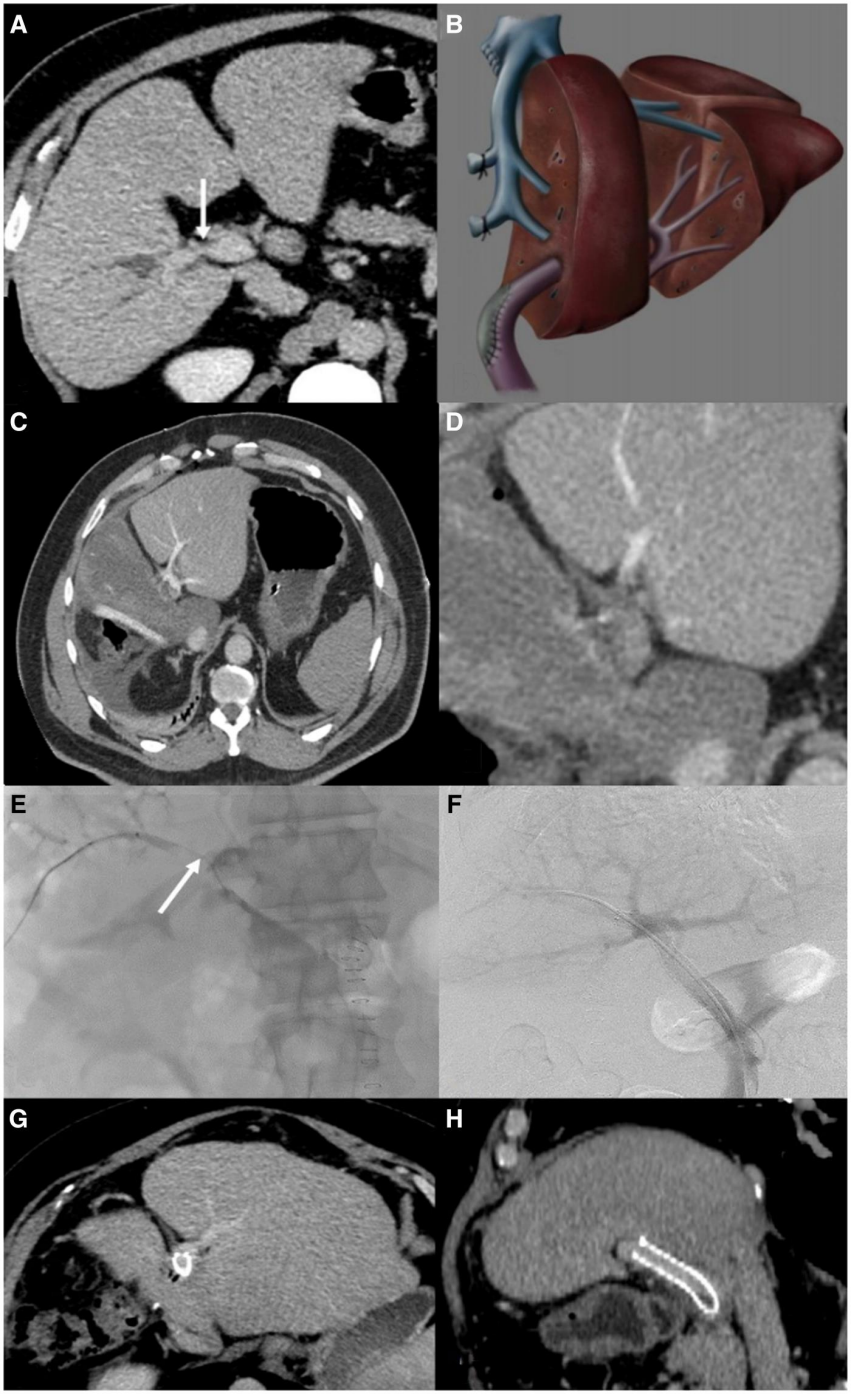
Review of the case. (A) Portal venous phase computerized tomography (CT) axial image showing the relationship between hilar cholangiocarcinoma and the portal vein (arrow). (B) The repair site of the portal vein shown by a hand drawing. (C and D) Axial images from the CT (portal venous phase) showing the postoperative portal vein thrombosis. (E) Fluoroscopy revealed portal vein occlusion (arrow). After catheterization, a valved infusion catheter was inserted for intravascular tPA infusion due to thrombus load in the portal vein. (F) Persistence of the occluded segment was observed following 24 hours’ tPA infusion, and this segment was stented (arrow). (G and H) At 18 months postoperatively, control portal venous phase axial and coronal CT images showed that the stent and intraparenchymal portal venous system were patent.

**Figure 2.**
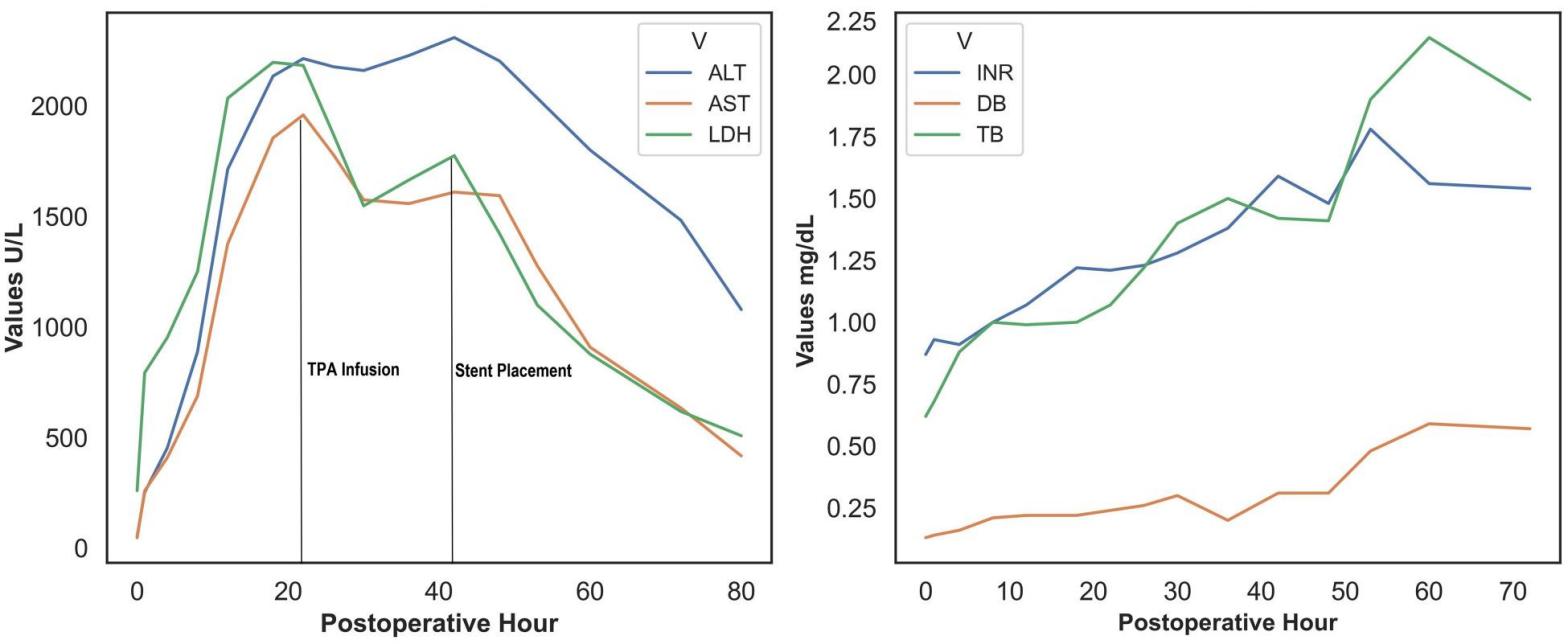
Biochemical alterations in the early postoperative period. Abbreviations: ALT = alanine aminotransferase; AST = aspartate aminotransferase; DB = direct bilirubin; INR = International normalized ratio; LDH = lactate dehydrogenase; TB = total bilirubin; tPA = tissue plasminogen activator.

A 55-year-old male who complained of abdominal pain and was admitted to our hospital. His blood biochemistry revealed alanine amninotrasferase; 52 U/L, aspartate aminotransferase; 47 U/L, gamma glutamyl trasferase; 726 U/L, alkaline phosphatase; 181 U/L, direct bilirubin; 0.13 mg/dL, and total bilirubin; 0.62 mg/day. Carbohydrate antigen 19-9, carcinoembryonic antigen and alpha fetoprotein levels were 19.9 U/mL, 2.92 µg/L, and 2 µg/L, respectively. Gastroscopy and colonoscopy were performed on the patient, and no pathological findings were detected. The dynamic abdominal CT scan showed an appearance compatible with HC (Figure 1A). Volumetric analysis revealed 40% remnant liver volume after right hepatectomy. Therefore, preoperative PV embolization was not performed. In operation, tumour invasion in the right side of PV bifurcation (2 cm long, <50% of PV circumference) was detected. Therefore, wedge resection of the PV bifurcation was preferred for R0 resection in addition to right hepatectomy, resection of the extrahepatic biliary tract, and regional lymph node dissection. The PV defect was repaired with a patch of an autologous umbilical vein graft (Figure 1B). After PV reconstruction, intraoperative Doppler USG was performed and it was observed that the portal flow was normal. Operative time was 300 min. Intermittent portal triad clamping was applied (cumulative clamping time 60 min-including PV reconstruction). The amount of bleeding during surgery was 1000 mL. Based on the International Study Group of Liver Surgery definition,^5^ grade B POHF developed on postoperative day (POD) 1. Computed tomography revealed acute PV thrombosis (Figure 1C and D). The left PV patent segment was cannulated from the distal part of the occlusion with a 21-gauge needle and 0.018″ guidewire and its followed by Coaxial 6F Sheath with RO Marker/4F dilator system insertion (AccuStick™ II Introducer System Boston Scientific, MA, United States) under ultrasound and fluoroscopy guidance. Fluoroscopy revealed segmental PV thrombosis (7 cm in length) (Figure 1E), and a Cragg-McNamara™ valved 20 cm infusion catheter (Medtronic, Minneapolis, MN, United States) was installed through the thrombotic segment. Following the placement of the infusion catheter, intravascular recombinant tissue plasminogen activator (tPA) infusion was initiated at 1 mg/h from the catheter. Following 24 h of tPA infusion, control fluoroscopy imaging revealed residual segmental occlusion of the PV (2 cm in length), probably representing the repair site on the PV. Deployment of the bare metallic stent into the PV was planned in order to resolve the segmental occlusion problem in the PV. The residual occluded segment was deployed with a 10 mm diameter and 8 cm length of self-expandable stent (The Epic™ stent Boston Scientific Corp., Natric, MA, United States) (Figure 1F). Intravenous heparin was infused (5000 units) during the stent placement. The percutaneous tract was embolized using glue/lipiodol mixture (%50). Anticoagulation was provided with oral clopidogrel loading (300 mg) and acetylsalicylic acid (100 mg) at the end of the stent placement and regiment continued for 3 months as dual antiplatelet treatment. Clinical and biochemical improvement in the POHF were observed, with the help of interventional treatment and medical support, 40 h postoperatively (Figure 2).^6^ The patency of the stent was also verified 3 days after placement using Doppler ultrasound. The patient also suffered from right pleural effusion and mild pneumonia in his postoperative course. Pathological examination revealed well-differentiated adenocarcinoma 2.5 × 1.5 cm in size with 15 reactive lymph nodes. Tumour tissue demonstrated perineural and lymphovascular invasion. Direct invasion of PV wall is also determined by both macroscopic and microscopic examination. The tumour was classified as stage 3A (T3, N0, M0) based on the eighth edition of the American Joint Committee on Cancer Staging Manual. The patient was discharged at POD 43 with oral clopidogrel 75 mg per day and acetylsalicylic acid 100 mg per day for 3 months period. At the end of the third postoperative months, the patient was followed with subcutaneous injection of enoxaparine sodium 60 mg twice a day up to the 12th postoperative months. Since that period, patient was followed with acetylsalicylic acid 100 mg per day. The patient also received radiotherapy (5040 cGy) and chemotherapy (capecitabine for 3 months). Patient has been followed 51 months so far without tumour recurrence, with patent flow through the stent, and mild portal hypertension (Figure 1G and H).

## Discussion

Portal vein reconstruction (PVR) is increasingly being used as there is mounting evidence that overall survival is not significantly different in comparison to patients who do not have PVR because they have less advanced disease. So, there is clear oncological benefit including high R0 resection rates, but also the perioperative morbidity/mortality associated with PVR appears in several studies to be no different to those who do not have PVR, although there is some controversy in this area.^1^^,^^2^

Problems in liver perfusion are an important cause of POHF, and the patency of inflow and outflow of the liver should be checked immediately. In the early period of the PV thrombosis after liver surgery, it is rational to re-exploration of the operative field, surgical thrombectomy and revision of portal flow. However, separation of the hepaticojejunostomy is mandatory for re-exploration of the PV in cases with HC and the tension on the PV will not be relieved after re-anastomosis of oedematous bilioenteric openings. Therefore, the timely application of minimal invasive interventions for re-establishment of portal flow would be an overcoming treatment option. Mechanical thrombectomy as a viable option mostly in combination with tPA in the PV setting.^7^ In our opinion, concerning of the iatrogenic rupture, mechanical thrombus aspiration, or balloon angioplasty was not performed to resolve the acute PV graft occlusion in the acute stage of repaired PV site.

The main concern regarding interventional thrombolytic treatment with tPA is bleeding from the raw surface of the liver. Our initial experience indicated that 10 mg of tPA can be administered without bleeding side-effects.^8^ Similarly, we did not encounter this complication in the current case, despite the cumulative tPA dose being increased to 24 mg. However, close follow-up of the patient’s haemodynamic status and intra-abdominal drains should be performed. In case of significant suspicion of re-occlusion of the PV, intravascular stent placement should be preferred as a viable solution based on the aetiology of the thrombosis.

## Learning points

- Postoperative portal vein (PV) thrombosis is a severe complication occurring in 2%-9% of patients requiring PV reconstruction and leads to postoperative hepatic failure and death in most cases.
- Despite PV repair, the patency of the repair site may not be guaranteed.
- The experience herein reported suggests that intraportal tPA and PV stenting with percutaneous approach can be reliable salvage treatment of acute PV thrombosis following PV repairing with a patch of an autologous umbilical vein graft.

## References

[1] Nagino M , Ebata T, Yokoyama Y, et al Evolution of surgical treatment for perihilar cholangiocarcinoma: a single-center 34-year review of 574 consecutive resections. Ann Surg. 2013;258:129-140. 23059502 10.1097/SLA.0b013e3182708b57

[2] Wu XS , Dong P, Gu J, et al Combined portal vein resection for hilar cholangiocarcinoma: a meta-analysis of comparative studies. J Gastrointest Surg. 2013;17:1107-1115. 23592188 10.1007/s11605-013-2202-9

[3] Bachellier P , Rosso E, Pessaux P, et al Risk factors for liver failure and mortality after hepatectomy associated with portal vein resection. Ann Surg. 2011;253:173-179. 21233614 10.1097/SLA.0b013e3181f193ba

[4] Mizuno T , Ebata T, Yokoyama Y, et al Combined vascular resection for locally advanced perihilar cholangiocarcinoma. Ann Surg. 2022;275:382-390. 32976284 10.1097/SLA.0000000000004322

[5] Rahbari NN , Garden J, Padbury R, et al Posthepatectomy liver failure: a definition and grading by the International Study Group of Liver Surgery (ISGLS). Surgery. 2011;149:713-724. 21236455 10.1016/j.surg.2010.10.001

[6] Ocak İ , Topaloğlu S, Acarli K. Posthepatectomy liver failure. Turk J Med Sci. 2020;50:1491-1503. 32718126 10.3906/sag-2006-31 PMC7605090

[7] Hollingshead M , Burke CT, Mauro MA, Weeks SM, Dixon RG, Jaques PF. Transcatheter thrombolytic therapy for acute mesenteric and portal vein thrombosis. J Vasc Interv Radiol. 2005;16:651-661. 15872320 10.1097/01.RVI.0000156265.79960.86

[8] Oguz S , Tayar S, Topaloglu S, et al Enhancing hepatic microcirculation in postoperative hepatic failure with intra-arterial recombinant tissue plasminogen activator treatment. Exp Clin Transplant. 2017;74. 10.6002/ect.2017.0074 29251575

